# The role of reactive oxygen species-mitophagy regulation in the treatment of osteoarthritis by active Chinese herbal medicine monomers: a review

**DOI:** 10.3389/fmed.2025.1686190

**Published:** 2025-11-03

**Authors:** Runjia Lei, Chaoqing Zhou

**Affiliations:** ^1^School of Traditional Chinese Medicine, School of Chinese Medicine, Macau University of Science and Technology, Macau, China; ^2^Third Department of Orthopedics and Traumatology, Zhuhai Hospital of Integrated Traditional Chinese and Western Medicine, Zhuhai, China

**Keywords:** osteoarthritis, Chinese herbal medicine monomers, reactive oxygen species, mitophagy, mitochondrion

## Abstract

The primary pathological features of osteoarthritis (OA) involve articular cartilage degradation and structural damage, coupled with osteophyte formation and inflammatory responses. As aging populations expand, the prevalence of knee osteoarthritis has risen substantially, severely compromising patients’ quality of life. Current therapeutic strategies for knee osteoarthritis remain limited in clinical efficacy, creating an urgent need for novel treatments that are both effective and safe. Chinese herbal medicine monomers have demonstrated significant potential in OA management, offering multi-pathway therapeutic effects, multi-target modulation, and favorable safety profiles. However, its underlying mechanisms require further elucidation. Mitophagy, a selective mitochondrial quality control mechanism that eliminates reactive oxygen species-damaged organelles, plays a crucial role in maintaining chondrocyte homeostasis and function. Emerging evidence highlights the regulatory significance of mitophagy in OA progression, presenting novel therapeutic perspectives. This review comprehensively analyzes the molecular mechanisms and physiological roles of the oxidative stress-mitophagy axis in knee osteoarthritis pathogenesis, while summarizing recent advances in herbal monomer-mediated regulation of this pathway. Future research directions are proposed to facilitate the systematic exploration and clinical translation of Chinese herbal medicine in OA therapeutics.

## 1 Introduction

Osteoarthritis (OA) represents a highly prevalent and etiologically heterogeneous degenerative joint disease, clinically manifested by progressive cartilage erosion, osteophytosis, and low-grade synovitis (1). The recalcitrant pain and substantial disability associated with OA profoundly compromise life quality of patients, while engendering considerable socioeconomic consequences (2). Contemporary management of OA encompasses physical modalities, pharmacological interventions, and surgical approaches, all principally directed toward symptomatic relief and functional preservation. Nevertheless, therapeutic efficacy remains constrained by iatrogenic complications of pharmacotherapy, suboptimal treatment adherence, and inherent perioperative risks (3). This therapeutic impasse underscores the critical need for elucidating OA pathogenesis to facilitate development of mechanism-based targeted therapies.

Monomeric compounds derived from Chinese herbal medicine, which have garnered increasing scientific attention, exhibit multidimensional therapeutic potential in addressing multifactorial pathologies such as osteoarthritis (4, 5). Their natural provenance, rooted in botanical evolution, inherently confers low systemic toxicity and superior biocompatibility, attributes that distinguish them from synthetic pharmaceuticals (6). Through advanced purification protocols, which systematically eliminate impurities and xenobiotic contaminants, these compounds achieve a safety profile that minimizes adverse reactions and mitigates cumulative risks inherent to chronic pharmacotherapy (7). Mechanistically, they operate through poly-pharmacological networks, not only targeting discrete pathological nodes but also orchestrating synergistic effects across divergent signaling pathways, thereby resolving the therapeutic limitations of single-target interventions in complex diseases (8, 9). Culturally, their alignment with principles of traditional Chinese medicine (TCM), which emphasize holistic healing, fosters patient adherence (10), while Chinese agricultural-industrial integration, a system that harmonizes large-scale medicinal cultivation with advanced technology-driven extraction, guarantees resource scalability and ecological sustainability (11). The translational paradigm bridging millennia-old empirical knowledge, as exemplified by antimalarial efficacy of artemisinin, with contemporary molecular dissection tools accelerates drug discovery timelines and delivers precision therapies that reconcile ancestral wisdom with cutting-edge innovation (12). Consequently, prioritizing monomers in osteoarthritis therapeutics, which currently rely on symptom-alleviating agents with iatrogenic risks, could redefine global standards by offering solutions that integrate biosafety, mechanistic versatility, and culturally resonant care. Nevertheless, the exact molecular cascades through which these compounds exert chondroprotective and anti-inflammatory effects remain incompletely mapped, necessitating further investigations.

## 2 The process and regulatory mechanism of mitophagy

### 2.1 Mitochondria are the main site of reactive oxygen species generation

The mitochondrial matrix, housing the tricarboxylic acid (TCA) cycle, establishes a microenvironment characterized by elevated partial oxygen pressure. This oxygen enrichment potentiates Reactive Oxygen Species (ROS) generation by providing abundant substrate for univalent electron transfer to molecular oxygen. Multiple pathological factors, including perturbations inΔψm (mitochondrial membrane potential), compromised electron transport chain (ETC) complex activity, or substrate deficiency, collectively increase electron leakage probability, thereby augmenting ROS formation through aberrant oxygen reduction (13). During oxidative phosphorylation, electrons normally undergo sequential transfer through ETC complexes I-IV before final reduction of oxygen to water. However, an estimated 0.2–2% of electrons deviate from this pathway, undergoing direct single-electron transfer to oxygen at complexes I and III, yielding superoxide anion (O_2_^−^) as the primary ROS byproduct (14–16). The inherent redox activity of iron–sulfur (Fe-S) clusters within ETC complexes presents an additional ROS source. These evolutionarily conserved cofactors, while essential for electron shuttling, exhibit thermodynamic instability when exposed to matrix oxygen, spontaneously generating ROS via Fenton-like reactions (17).

### 2.2 The process of mitophagy

As the principal bioenergetic powerhouses of eukaryotic cells, mitochondria orchestrate adenosine triphosphate (ATP) biogenesis while governing critical physiological pathways whose dysregulation precipitates cellular dysfunction. This indispensable role necessitates stringent quality control mechanisms to maintain mitochondrial homeostasis (18). Mitophagy, a phylogenetically conserved selective autophagic process, serves as the cornerstone of mitochondrial quality surveillance by selectively eliminating dysfunctional organelles. The canonical mitophagic cascade progresses through four mechanistically distinct phases: (i) stimulus-induced mitochondrial depolarization triggering ubiquitin signaling cascades; (ii) LC3-mediated autophagosomal encapsulation forming double-membrane mitophagosomes; (iii) SNARE-dependent lysosomal fusion generating autolysosomes; and (iv) cathepsin-mediated proteolytic degradation enabling metabolic recycling (19).

### 2.3 ROS serves as the core initiating link of mitophagy

Mitophagy operates through two evolutionarily conserved molecular recognition paradigms: the ubiquitin-mediated degradation pathway and the receptor-dependent direct recognition pathway, each executing distinct yet complementary roles in mitochondrial quality surveillance. The canonical ubiquitin-dependent mechanism orchestrates mitochondrial clearance through sequential activation of the PINK1-Parkin signaling axis. PINK1, a mitochondrial-targeted serine/threonine kinase, exhibits dynamic subcellular localization. In normal mitochondria, due to the normal mitochondrial membrane potential (Δψm), PINK1 is imported into the mitochondrial matrix and degraded by proteases. When mitochondria are damaged (e.g., due to abnormal function of electron transport chain complexes III/IV), the production of ROS increases, which further impairs the integrity of the mitochondrial membrane and leads to a decrease in Δψm. As a result, PINK1 fails to enter the matrix and instead accumulates and becomes activated on the outer mitochondrial membrane (OMM) (20). The activated PINK1 phosphorylates ubiquitin molecules on the OMM, generating “phosphorylated ubiquitin (p-Ub).” This phosphorylation cascade induces the translocation of Parkin, which in turn mediates the polyubiquitination of substrates on the OMM (21). This modification is primarily achieved through K63-linked ubiquitin chains, which act as molecular signals to recruit autophagic adaptor proteins (such as p62/SQSTM1, NBR1, and OPTN) that possess both ubiquitin-binding domains and LC3-interacting regions (LIRs). The finally formed ubiquitin-LC3 scaffold initiates phagophore assembly, ultimately leading to the degradation of dysfunctional mitochondria by autolysosomes (22).

Conversely, the ubiquitin-independent pathway employs a cadre of OMM-resident LIR-containing receptors (BNIP3L/NIX, FUNDC1, BCL2L13) that bypass ubiquitination by directly engaging LC3/GABARAP proteins through their LIR motifs. These molecular sentinels detect mitochondrial stress signals and initiate selective encapsulation through LIR-mediated interactions with nascent autophagosomal membranes (23, 24). Notably, as a key direct driver, ROS can precisely mediate various oxidative modifications of protein cysteine residues, including disulfide bond formation, S-glutathionylation, S-nitrosylation, and S-sulfinylation, which can directly regulate the dynamic changes in protein conformation, thereby achieving “switch-like” precise regulation of the function of target proteins (25, 26). Previous studies have shown that the mitophagy receptor FUNDC1 exhibits significant changes in protein modification activity during its activation process. For instance, in HeLa cells treated with hypoxia (1% O_2_) or the mitochondrial uncoupler FCCP (which disrupts mitochondrial membrane potential), a marked induction of dephosphorylation at serine 13 (Ser13) of FUNDC1 can be observed (27). However, no studies have clearly reported a direct association between the activation mechanism of mitophagy receptors (such as FUNDC1) and ROS-mediated modifications so far. Therefore, future research should prioritize exploring how ROS-driven oxidative modifications specifically regulate mitophagy receptor activation and their underlying mechanisms, a direction of significant scientific value.

Both pathways ultimately converge on lysosomal degradation via conserved membrane fusion machinery (For example, STX17-SNAP29-VAMP8 complex), yet their divergent initiation mechanisms provide layered protection against mitochondrial dysfunction (28). This dual-pathway architecture ensures robust mitochondrial homeostasis by integrating multiple damage sensors with the core autophagy machinery, thereby maintaining cellular metabolic integrity under varying stress conditions.

### 2.4 Reverse feedback regulation of mitophagy on ROS

**Figure 1:**
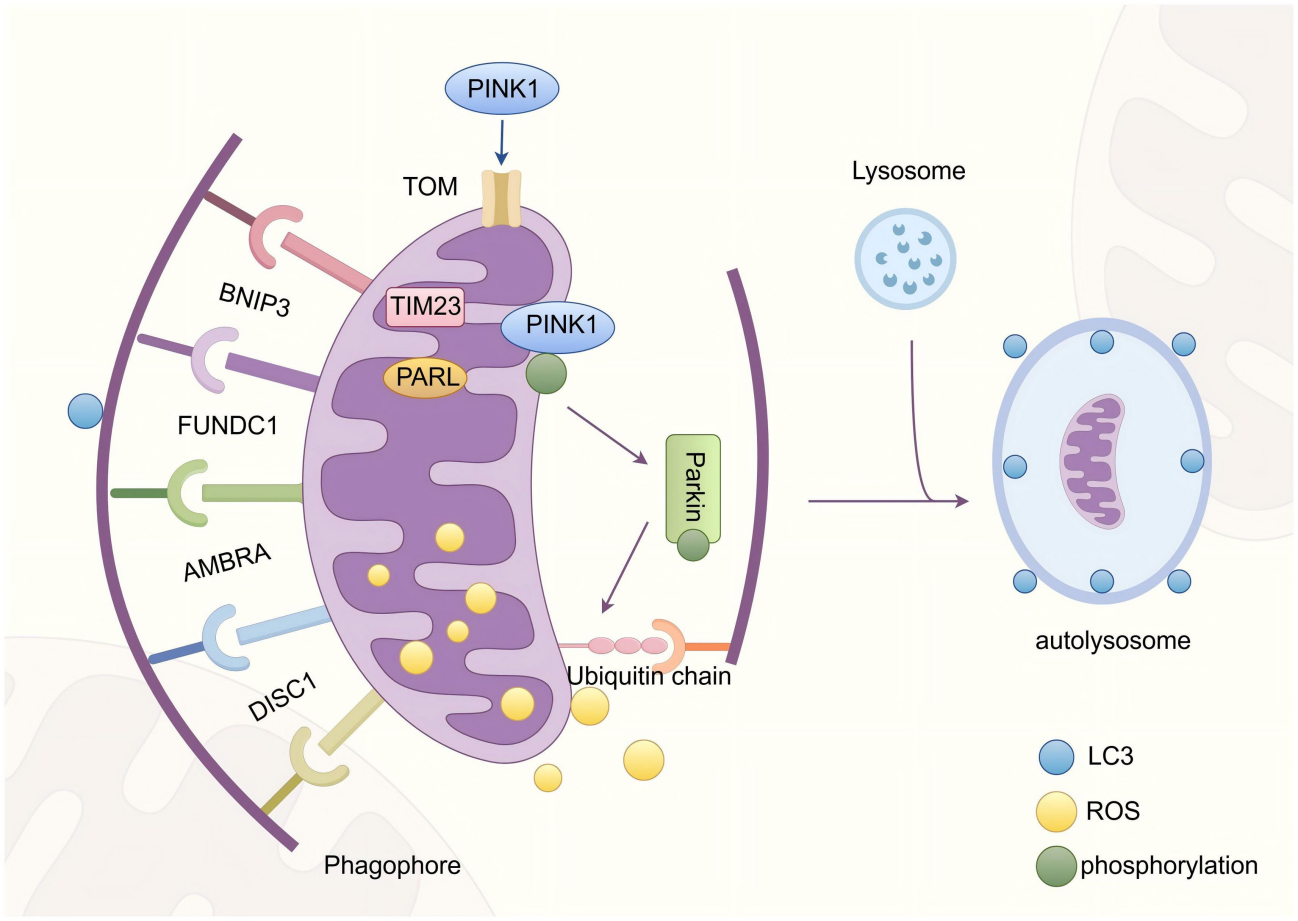
A brief overview of mitophagy.

Dysfunctional mitochondria constitute intracellular “hotspots” for ROS generation, wherein these aberrant organelles not only produce excessive ROS autonomously but also inflict damage upon adjacent healthy mitochondria, thereby establishing a self-perpetuating vicious cycle (29). The core mechanistic function of mitophagy resides in its capacity to precisely identify, sequester, and degrade such compromised mitochondria through lysosomal degradation. This elimination of primary ROS sources enables mitophagy to directly and efficiently attenuate global intracellular ROS concentrations (30). Following the removal of damaged mitochondria, cells initiate a mitochondrial biogenesis program which is primarily regulated by PGC-1α, generating functionally intact organelles to maintain mitochondrial homeostasis (31). The resultant robust mitochondrial network demonstrates enhanced electron transport efficiency coupled with diminished electron leakage, a combination that fundamentally suppresses ROS production at its origin (Figure 1).

## 3 ROS-mitophagy network in osteoarthritis

Articular cartilage represents a paradigm of metabolically constrained tissue due to its absence of vascular, lymphatic, and neural networks. Under homeostatic physiological conditions, this tissue exhibits minimal proliferative capacity, with cellular survival exclusively dependent upon passive nutrient diffusion (32). To accommodate the distinctive avascular high-load-bearing microenvironment, chondrocytes establish an unconventional metabolic paradigm wherein, even when cultured *in vitro* under oxygen-replete conditions, approximately 85–90% of their energy derives from anaerobic glycolysis, characterized by lactic acid generation through glucose catabolism, while mitochondrial oxidative phosphorylation contributes merely 10–15% (33). Notably, upon completion of their proliferative and differentiation phases during embryonic development, these cells transition into a quiescent state during adulthood. This terminal phenotype manifests as profoundly diminished proliferative activity, alongside an irreversible loss of multipotent differentiation potential (34, 35).

To overcome survival challenges inherent to their biological constraints, chondrocytes must sustain homeostasis through augmented autophagic activity wherein mitophagy constitutes the fundamental mechanism that preserves mitochondrial structural and functional integrity (36). When mitophagy dysfunction arises, damaged organelles accumulate which disrupts cellular energy equilibrium, thereby exacerbating chondrocyte dysfunction and accelerating cartilage degeneration (37). Specifically, such accumulated mitochondrial damage directly suppresses aerobic respiration, substantially diminishing ATP production via a pathway that merely functions as an auxiliary energy source for these cells; this energy deficit becomes particularly critical during high-demand states including injury repair or extracellular matrix synthesis (38). Concurrently, diminished mitophagic flux induces ROS accumulation, which elevates matrix metalloproteinase activity while suppressing type II collagen and aggrecan biosynthesis, a dual perturbation whose remediation through induced mitophagy has been experimentally demonstrated to restore extracellular matrix homeostasis and enhance cartilage biomechanical properties (39, 40). Furthermore, ROS synergizes with mitochondrial DNA released from impaired mitochondria to activate the NLRP3 inflammasome (41), triggering chondrocytes and synovial cells to secrete pro-inflammatory cytokines, such as interleukin-1β, tumor necrosis factor-α, and interleukin-6, thereby establishing an autocrine-paracrine inflammatory network that recruits infiltrating immune cells, polarizes them toward pro-inflammatory phenotypes (42, 43), and amplifies joint cavity inflammation which directly drives cartilage destruction (44). Mitophagy counteracts this cascade by sequestering compromised mitochondria, limiting damage-associated molecular pattern leakage, and restraining inflammasome hyperactivation (45). Simultaneously, elevated ROS upregulate transient receptor potential vanilloid 1 (TRPV1) expression, increasing nociceptive neuron sensitivity that directly contributes to osteoarthritis pain pathogenesis (46), a mechanism corroborated by clinical evidence showing significant positive correlation between synovial fluid reactive oxygen species levels and visual analog scale pain scores (47).

**Figure 2:**
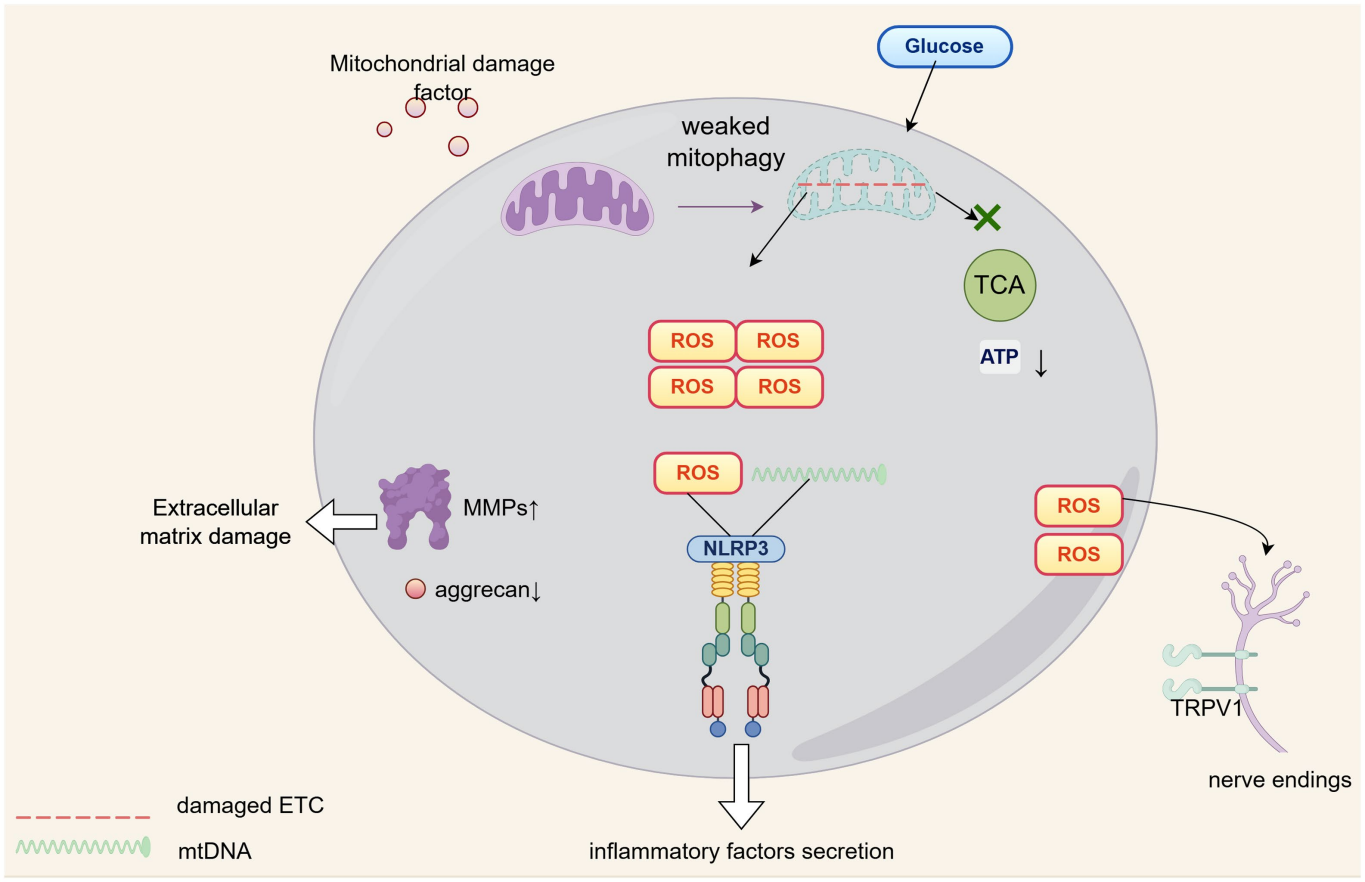
The role of ROS-mitophagy in OA progression.

A schematic diagram depicting the molecular network of the ROS-mitophagy axis in the context of OA pathophysiology is shown in Figure 2.

## 4 The mechanism of action of monomers of Chinese herbal medicine in targeted regulation of mitophagy for the prevention and treatment of OA

### 4.1 Inhibiting cellular oxidative stress

Mitochondrial dysfunction and ferroptosis induced by iron overload are key triggers for cartilage damage in OA (48), and a variety of natural compounds exert protective effects by targeting this pathway. Cardamonin, derived from *Alpinia katsumadai* Hayata, alleviated iron overload-induced mitochondrial dysfunction by restoring membrane potential, reducing ROS and intracellular Fe^2+^ levels, and preventing ultrastructural damage characterized by mitochondrial cristae fragmentation and outer membrane rupture, whose therapeutic efficacy was further linked to the upregulation of p53 and GPX4 (49). Biochanin A, serves as a pharmacologically active ingredient of *Trifolium pratense* L., directly targeted Nrf2/system xc^−^/GPX4 signaling pathway to scavenge ROS and prevent lipid peroxidation (50). Arctiin, from *Arctium lappa* L., enhanced chondrocyte viability, inhibited chondrocyte apoptosis and ferroptosis, reduced intracellular iron, ROS and lipid ROS levels, repaired mitochondrial damage, and finally alleviated chondrocyte oxidative stress by activating the AKT/NRF2/HO-1 signaling pathway (51). Naringenin, further demonstrated iron-chelating properties, upregulating Nrf2 and HO-1 to counteract iron overload-associated cartilage damage (52). And the chondroprotective efficacy of Ruscogenin arised from two elucidated pharmacological actions, which diminished ROS levels within chondrocytes to suppress ferroptosis, while concurrently targeted sirtuin 3 (SIRT3) to modulate macrophage reprogramming, thereby ameliorating joint inflammatory microenvironments (53).

Loganin, a bioactive compound derived from *Cornus officinalis* Siebold & Zucc, demonstrated protective efficacy against osteoarthritis through attenuating cartilage degradation and osteophyte formation, thereby delaying disease progression. This effect was mediated via reduction of ROS within chondrocytes, suppression of NLRP3 inflammasome activation, and potentiation of the NRF2/HO-1 signaling axis (54). It is worth noting that in this study, the interaction between Loganin and Nrf2 was confirmed by molecular docking, and immunofluorescence experiments verified that Loganin exerts its antioxidant effect by promoting the nuclear translocation of Nrf2. Ginkgolide C, pharmacologically active ingredient of *Ginkgo biloba*, inhibited NLRP3 inflammasome activation through modulation of ROS-mediated phospho-IRE1α suppression and Nrf2 pathway induction, which collectively alleviated joint pain in osteoarthritic rats, exert anti-inflammatory actions, impede extracellular matrix catabolism, and confer chondroprotection (55). Comparable pharmacological properties characterized nodakenin, the principal coumarin constituent of *Radix Angelicae biseratae*, wherein mitochondrial Drp1/ROS/NLRP3 axis regulation improved subchondral bone architecture, mitigates cartilage deterioration, and reduced knee joint inflammation in murine models (56). Cardamonin, isolated from fruits of chasteberry plant, exhibited documented therapeutic potential in knee osteoarthritis according to Li et al., operating through SIRT1 expression upregulation coupled with p38MAPK pathway inhibition that suppressed NLRP3 inflammasome vesicle formation and reversed iron overload-induced chondrocyte apoptosis alongside cartilage degeneration (57). Perillaldehyde, a compound extracted from *Perilla frutescens*, can alleviate IL-1β-induced mitophagy-associated apoptosis of chondrocytes and NLRP3-mediated inflammatory response by downregulating the expression of ALOX5 and inhibiting the activation of NF-κB signaling pathway, thus exhibiting potential protective effects in OA (58). Collectively, these studies substantiate that naturally occurring phytochemicals alleviate cartilage degradation and decelerate osteoarthritis progression by diminishing ROS burden, blocking NLRP3 inflammasome activation, and concurrently activating antioxidant defense systems exemplified by the Nrf2 pathway or modulating mitochondrial functional cascades such as the Drp1/ROS/NLRP3 axis.

In addition to inducing inflammatory responses and ferroptosis, ROS can further exacerbate mitochondrial damage and amplify mitochondrial dysfunction (59), while natural compounds can regulate this pathway through multiple targets. Apple polyphenols mitigated mitochondrial oxidative stress in OA by enhancing mitochondrial dehydrogenase activity and superoxide dismutase (SOD) bioavailability, which suppressed ROS-mediated inflammatory pathways, thereby attenuating synovitis and cartilage erosion facilitate by MMP-13 (60). Ginsenoside Rb1, isolated from *Panax ginseng* C., ameliorated oxidative stress through suppression of reactive oxygen species generation while concurrently preserving mitochondrial integrity and exerting articular cartilage protective effects. Within osteoarthritic conditions, this compound reduced reactive oxygen species production in a time-dependent manner, thereby significantly inhibiting prostaglandin E2 and matrix metalloproteinase-3 synthesis (61). Dendrobine, an alkaloid from *Dendro bium nobile* Lindl., rescued IL-1β-induced mitochondrial dysfunction by normalizing ROS overproduction and enhancing respiratory chain activity (62). Isoorientin, from both *Gentiana straminea* Maxim. and *Fagopyrum esculentum* Moench, modulated mitochondrial membrane potential and SOD/MDA ratios by regulating MAPK and PI3K/Akt pathways, offering protection against oxidative stress-driven chondrocyte death (63). Oleanolic acid, an organic acid derived from *olea europaea* L., attenuated synovial inflammation by elevating SIRT3 expression and inhibiting NF-κB nuclear translocation. Its pleiotropic actions included ROS neutralization, COX-2/PGE2 suppression, and restoration of mitochondrial bioenergetics (64).

As a core antioxidant defense system, the Nrf2 pathway is a key target for natural compounds to exert OA-protective effects (65). Beyond the aforementioned studies, multiple studies have confirmed that Chinese medicine monomers can target Nrf2 to promote downstream cellular antioxidant enzymes such as HO-1, enhance cellular antioxidant capacity to scavenge excessive ROS in OA, and simultaneously inhibit ROS-mediated inflammatory pathways, reduce chondrocyte apoptosis, and ultimately alleviate the pathological progression of OA. Valencene, a sesquiterpenoid compound extracted from *Cyperus rotundus*, is widely used in food flavors. Chen et al. revealed that it can scavenge ROS by activating the NRF2/HO-1 pathway, and reduce inflammation and cartilage matrix degeneration by inhibiting the NF-κB and MAPK signaling pathways (66). A subsequent integrated traditional Chinese and Western medicine study pointed out that the combined use of curcumin and catalase can synergistically enhance ROS scavenging capacity and inhibit oxidative stress-induced chondrocyte damage and apoptosis. The mechanism lies in the synergistic effect of curcumin regulating the NRF2/HO-1 pathway to upregulate antioxidant enzymes and catalase degrading hydrogen peroxide (67). In addition, although irisin does not directly rely on the Nrf2 pathway, it can indirectly inhibit inflammation-mediated oxidative stress and insufficient synthesis of chondrocyte extracellular matrix by maintaining mitochondrial biogenesis, dynamics, and autophagic processes. Together with Nrf2 pathway-targeting compounds, it forms a multi-dimensional oxidative stress regulatory network, providing a comprehensive theoretical basis for the treatment of OA with natural products (68).

### 4.2 Activating the PINK1-PARKIN signaling pathway

Curcumin, a classic traditional Chinese medicine monomer derived from a variety of plants, enhanced mitochondrial functionality in osteoarthritic chondrocytes through targeted mitophagy induction. Treatment with curcumin restores mitochondrial membrane potential, reduces ROS and intracellular Ca^2+^ overload, and elevates ATP synthesis. Quantitative proteomic analyses confirmed its reliance on the AMPK/PINK1/Parkin axis, with upregulated expression of phosphorylation AMPK PINK1, Parkin, and microtubule-associated protein 1 light chain 3 beta (LC3B). These coordinated actions decelerate OA progression by resolving mitochondrial dysfunction and oxidative stress (69). Protocatechuic aldehyde, a phenolic compound isolated from *Salvia miltiorrhiza* rhizomes, delayed chondrocyte senescence via PINK1/Parkin pathway potentiation. Notably, it elevated LC3-II/LC3-I ratios and enhanced Parkin/PINK1 colocalization at depolarized mitochondria, driving selective autophagic clearance of damaged organelles. This mechanism not only reinforced mitochondrial turnover but also mitigated age-related chondrocyte dysfunction, positioning it as a novel therapeutic candidate for OA management (70).

Acetyl zingerone is an active derivative found in *Zingiber officinale* Roscoe. It exhibited biological activities such as anti-inflammatory and antioxidant properties. By directly activating the PINK1/Parkin signaling pathway, it could promote mitophagy, thereby inhibiting chondrocyte pyroptosis and alleviating the progression of osteoarthritis (71). Koumine possessed pharmacological activities such as anti-inflammation and analgesia. By activating PINK1/Parkin-mediated mitophagy, it could inhibit IL-1β-induced chondrocyte inflammation, and ultimately reduced the extracellular matrix degradation in osteoarthritic cartilage (72).

Lentinan, the primary therapeutically active constituent derived from *Lentinus edodes*, demonstrated capacity to attenuate interleukin-1β-induced extracellular matrix degradation and inflammatory factor secretion in chondrocyte cultures when administered *in vitro*, while concurrently inhibiting cartilage degeneration in osteoarthritic rat models during *in vivo* application. The mechanism underlying these protective effects involved enhanced mitophagy promotion and mitochondrial homeostasis maintenance, which operated through mTOR-mediated modulation of the PINK1/Parkin pathway (73). Similarly, ginsenoside Rh1exhibited inhibitory actions against osteoarthritis progression and chondrocyte apoptosis both *in vivo* and *in vitro*, which was achieved through AMPK-dependent regulation of PINK1/Parkin-directed mitophagy, thereby preserving chondrocyte integrity across experimental contexts (74).

### 4.3 Activating non-ubiquitination-dependent mitophagy

Artemisinin, a flavonoid compound isolated from *Artemisia annua* L., enhanced autophagic capacity in knee osteoarthritis chondrocytes through modulation of the PI3K/Akt/mTOR signaling pathway. Experimental evidence indicated that this phytochemical upregulates expression of autophagy-related protein 5 (ATG5), autophagy effector protein Beclin-1, and microtubule-associated protein LC3, while promoting LC3-II conversion and mitochondrial activation. Consequently, artemisinin significantly ameliorated arthritis symptoms in rodent models (75). Similarly, Baicalin, from *Scutellaria baicalensis* Georgi, elevated expression of anti-apoptotic protein Bcl-2 while suppressing pro-apoptotic proteins Bax and Caspase-3. Mechanistically, these glucosides enhanced Beclin-1 and LC3II/I expression, reduced p62 accumulation, and restored mitochondrial membrane potential. By reversing interleukin-1β-mediated dysregulation of the PI3K/Akt/mTOR axis, they coordinate autophagy induction with mitochondrial functional preservation, thereby exerting chondroprotective effects (76).

Dihydromyricetin, a natural SIRT3 agonist derived from *Ampelopsis grossedentata*, rebalanced mitochondrial apoptosis and mitophagy. By activating SIRT3, DHM suppresses mitochondrial apoptosis via downregulation of Bax and upregulation of Bcl-2, effectively attenuating chondrocyte apoptosis (77). Oleanolic acid demonstrated multifaceted therapeutic potential in OA. It inhibited Caspase-3 expression while augmenting autophagosome and autolysosome formation via PI3K/Akt/mTOR pathway suppression. Oleanolic acid significantly reduced PI3K, p62, Akt, and mTOR protein levels and phosphorylation states, concomitant with elevated Beclin-1 and LC3-II/LC3-I ratios. Additionally, Oleanolic acid downregulated pro-inflammatory mediators (iNOS, COX-2) and matrix-degrading enzymes (MMP-3, MMP-13), ameliorating both inflammatory cascades and extracellular matrix catabolism (78).

At present, there are no relevant reports indicating that Chinese herbal monomers can initiate mitophagy through mitophagy receptors such as FUNDC1 or BNIP3L. Notably, in prior research on herbal compound formulas, Yang et al. first characterized the composition of a specific formula, then employed transcriptomic analyses to demonstrate that Gubi Zhitong Formula (GBZTF) attenuated OA progression by activating mitophagy pathways. Subsequent cellular experiments further confirmed that GBZTF induced mitophagy via BNIP3L regulation (79). Consequently, this study suggests that future investigations, while continuing to focus on the PINK1-Parkin pathway, should prioritize elucidating the role of ubiquitin-independent mitophagy in mediating the therapeutic effects of Chinese herbal monomers on OA.

A summary of the mechanisms by which Chinese herbal medicine monomers targeted regulation of mitophagy prevented OA was shown in Table 1.

**Table 1:** Mechanism of action of Chinese herbal medicine monomers in treating osteoarthritis via targeted regulation of mitophagy.

Monomers	Origin	Model	Mitophagy-relative mechanism	References
Cardamonin	*Alpinia katsumadai* Hayata	Primary chondrocytes from rats and rat OA model built by anterior cruciate ligament transaction	Reducing ROS and intracellular Fe^2+^ levels by upregulating p53 and GPX4	(49)
Biochanin A	*Trifolium pratense* L.	Surgically-induced OA model mice and chondrocytes	Scavenge ROS and prevent lipid peroxidation by Nrf2/system xc^−^/GPX4	(50)
Arctiin	*Arctium lappa* L.	Rat OA model established by destabilized medial meniscus	Alleviates oxidative stress in chondrocytes via AKT/NRF2/HO-1 signaling pathway	(51)
Naringenin	*Amacardi-um occidentale L*.	Iron dextran and surgery-induced destabilized medial meniscus rats and chondrocytes	upregulated Nrf2 and HO-1 to anti-oxidative stress	(52)
Ruscogenin	*Ophiopogon japonicus* (L.f.) Ker Gawl.	Rats with anterior cruciate ligament transection-induced osteoarthritis and SW1353 cells	Regulate macrophage reprogramming by targeting Sirt3 and attenuate the ROS level	(53)
Loganin	*Cornus officinalis* Siebold & Zucc	OA mouse model by performing medial meniscus destabilization surgery	Inhibits the ROS-NLRP3-IL-1β axis by activating the NRF2/HO-1 pathway	(54)
Ginkgolide C	*Ginkgo biloba*	Monosodium Iodoacetate-induced osteoarthritis rat model	Inhibited activation of NLRP3 inflammasome by restraining ROS-mediated p-IRE1α and activating Nrf2/NQO1	(55)
Nodakenin	*Radix Angelicae biseratae*	OA mice by destabilized medial meniscus and primary chondrocytes	Regulate mitochondrial Drp1/ROS/NLRP3 axis	(56)
Cardamonin	Chasteberry plant	Iron overload mouse model and then surgically induced osteoarthritis, primary chondrocytes	Attenuate ROS production and NLRP3 inflammasome activation via the SIRT1/p38MAPK signaling pathway	(57)
Perillaldehyde	*Perilla frutescens*	IL-1β-treated chondrocytes and destabilized medial meniscus induced rats	Inhibit mitophagy-associated apoptosis and NLRP3-mediated inflammation by regulating ALOX5/NF-kB signaling	(58)
Apple polyphenols	*Malus pumila Mill*	Surgically-induced OA model rats and HIG-82 synoviocytes	Suppress oxidative stress by enhanced SOD activity	(60)
Quercetin	Various plants	LPS treated C28/I2 cell and partial medial meniscectomy-treated Wistar rats	Reduced MDA levels, and activated the Keap1/Nrf2 signaling axis	(61)
Ginsenoside Rb1	*Panax ginseng* C. A. Mey.	Rabbit osteoarthritis induced by hollow trephine on the femur trochlea	Reduced intracellular ROS through down-regulation of p-Akt, p-P38, and p-P65	(62)
Dendrobine	*Dendro bium nobile* Lindl.	OA rats model constructed by the transection of anterior cruciate ligament and primary chondrocytes from rats	Improved mitochondrial function and reduced intracellular ROS via NF-κB	(63)
Isoorientin	*Gentiana straminea* Maxim. and *Fagopyrum esculentum* Moench	H_2_O_2_ induced chondrocytes	Modulate mitochondrial membrane potential and SOD/MDA ratios by regulating MAPK and PI3K/Akt pathways	(64)
Oleanolic acid	*Olea europaea* L.	Fibroblast-like synoviocytes	Included ROS neutralization via the SIRT3-NF-κB axis	(65)
Valencene	*Cyperus rotundus L.*	IL-1β induced primary chondrocytes and mouse model constructed by destabilization of medial meniscus	Reverse the rise of ROS by NRF2/HO-1/NQO1 pathway and downstream phosphorylation of NFκB P65	(67)
Curcumin	Various plants	Anterior cruciate ligament transection of the right knee for rats and primary chondrocytes	Regulate the NRF2/HO-1 pathway to scaveng ROS	(68)
Curcumin	Various plants	Sodium monoiodoacetate-induced rat OA model and IL-1β induced OA chondrocyte	Maintained mitochondrial homeostasis and promoted PINK1/Parkin expression	(70)
Protocatechuic aldehyde	*Salvia miltiorrhiza* rhizomes	Medial meniscus -induced mice OA model and LPS induced chondrocyte	Facilitated mitochondrial autophagy by PINK1/Parkin pathway	(71)
Acetyl zingerone	*Zingiber officinale* Roscoe	Medial meniscus destabilization mice and LPS plus ATP induced ATDC5	Activated the PINK1/Parkin	(72)
Koumine	*Gelsemium elegans* (Gardn. & Champ.) Benth.	IL-1β induced RCCS-1 and rat OA model established by intra-articular injection of 2% papain	Activated PINK1/Parkin-mediated mitophagy and promoted LC3II/I	(73)
Lentinan	*Lentinus edodes*	IL-1β induced chondrocytes and rat OA model with anterior cruciate ligament transection	Activate mitophagy via mTOR/PINK1/Parkin pathway	(74)
Ginsenoside Rh1	*Panax ginseng* C. A. Mey.	IL-1β induced chondrocytes and rat OA model with anterior cruciate ligament transection	Attenuates chondrocyte senescence via AMPK/PINK1/Parkin-mediated mitophagy	(75)
Artemisinin	*Artemisia annua* L.	IL-1β stimulated chondrocytes and destabilized medial meniscus rats	Promoted the conversion of LC3-II and the activation of mitochondria by PI3K/AKT/mTOR signaling	(76)
Baicalin	*Scutellaria baicalensis* Georgi	IL-1β stimulated chondrocytes	Enhanced Beclin-1 and LC3II/I expression, reduce p62 accumulation, and upregulated Pink1	(77)
Dihydromyricetin	*Ampelopsis grossedentata*	Chondrocytes and post-traumatic OA model	Recouped endogenous mtApoptosis and mitophagy balance by SIRT3	(78)
Oleanolic acid	*Olea europaea* L.	Rat OA model established by intra-articular injection of monosodium iodoacetate and IL-1β induced ATDC5 cell	Inhibited the PI3K/AKT/mTOR signaling pathway and elevated LC3-II/LC3-I ratios	(79)

## 5 Limitations of the current research

Although there are currently many studies on Chinese herbal medicine treating osteoarthritis by improving mitophagy, most of the current research focuses on basic experiments, and there is a lack of high-quality and high-level clinical studies. At the same time, the current research lacks the verification of the direct interaction between the monomers of Chinese herbal medicine and target molecules (such as Parkin/PINK1, Bcl-2). Most of the studies mainly focus on regulating a single mitochondrial-related pathway, and the detection indicators are limited. However, various mitochondrial-related pathways and targets may affect the development and outcome of osteoarthritis through interactions, and it is also worthy of in-depth investigation whether the changes in mitochondria are related to the alterations in mechanisms such as histones and the endoplasmic reticulum. Finally, as the center of energy metabolism, mitochondrial dysfunction can lead to changes in metabolic activity and the production of metabolic by-products such as lactic acid, and lactic acid can serve as a substrate for lactylation modification to participate in protein modification (80). Therefore, subsequent research can further focus on the roles of mitophagy and lactylation modification in osteoarthritis and the protective mechanisms of Chinese herbal medicine against these processes.

## 6 Summary and prospects

In recent years, mounting evidence has substantiated the pivotal role of bioactive monomers derived from Chinese herbal medicine in mitigating osteoarthritis through modulation of the ROS-mitophagy axis. These phytochemicals effectively attenuate oxidative stress by suppressing NADPH oxidase activity and activating the Nrf2-mediated antioxidant pathway, while concurrently orchestrating mitophagy-related signaling cascades to facilitate the selective removal of dysfunctional mitochondria, thereby preserving chondrocyte homeostasis and decelerating OA progression. Unlike conventional antioxidants, TCM monomers possess distinctive advantages, including multi-target regulatory capacity, favorable biosafety profiles, and natural origins. However, their precise molecular mechanisms, dose–response relationships, and discrepancies between *in vitro* and *in vivo* findings warrant further elucidation.

Future investigations should prioritize the following directions: (1) Target identification represented by activity-based protein profiling (81), limited proteolysis-coupled mass spectrometry (82) to delineate the interactions between TCM monomers and ROS/mitophagy-associated proteins; (2) Real-time spatiotemporal monitoring, for example live-cell imaging coupled with ROS/autophagosome biosensors, to dynamically track intracellular redox and mitophagic flux; (3) Clinical translation strategies (e.g., synergistic therapy with metformin, quantification of synovial fluid mtDNA as a mitophagy biomarker) and (4) Advanced drug delivery optimization, take hyaluronic acid-functionalized nanocarriers as an example to enhance joint-specific biodistribution.

Recent years have witnessed growing academic interest in plant-derived exosome-like nanoparticles (PELNs) as an innovative dosage form for traditional Chinese medicine. These nanovesicles exhibit significant advantages including broad source availability, low immunogenicity, and high accessibility. Substantial evidence demonstrates that PELNs possess multifaceted pharmacological properties encompassing anti-inflammatory, anti-tumor, and immunomodulatory activities, which render them therapeutically valuable against malignancies, inflammatory disorders, and bacterial infections (83, 84). A pioneering study concerning osteoarthritis therapy recently engineered a supramolecular network hydrogel by integrating rhein, a bioactive TCM monomer, with Spirulina platensis-derived exosomes. This composite system (designated Rh Gel@SP-EVs) concurrently delivers anti-inflammatory effects and metabolic support. Mechanistically, it attenuates pathological processes through selective inhibition of the JAK/STAT signaling pathway, thereby significantly suppressing aberrant reactive oxygen species generation and mitochondrial dysfunction within inflamed chondrocytes (85). Future investigations should prioritize three strategic directions: diversifying PELN sources and therapeutic applications, systematically screening anti-osteoarthritic PELNs from additional medicinal plants, and conducting comparative analyses of bioactive constituents and synergistic mechanisms across varied PELN types.

Moreover, artificial intelligence (AI)-assisted mining of TCM monomer libraries and their crosstalk with the ROS-mitophagy network, complemented by mechanistic reinterpretation of TCM theories through contemporary biomedical paradigms, will unveil novel therapeutic avenues for OA. Such interdisciplinary integration is poised to catalyze innovation in OA pharmacotherapy and foster convergence between traditional and modern medicine.
